# Investigating livestock management in the early Neolithic archaeological site of Cabecicos Negros (Almería, Spain) from the organic residue analysis in pottery

**DOI:** 10.1038/s41598-023-31036-6

**Published:** 2023-03-23

**Authors:** N. Tarifa-Mateo, M. Saña, X. Clop, A. Rosell-Melé, M. D. Camalich-Massieu, D. Martín-Socas

**Affiliations:** 1 Department of Prehistory, Autonomous University of Barcelona, 08193 Bellaterra, Spain; 2 Institute of Environmental Science and Technology (ICTA-UAB), Autonomous University of Barcelona, 08193 Bellaterra, Catalonia Spain; 3 Catalan Institution for Research and Advanced Studies (ICREA), 08010 Barcelona, Catalonia Spain; 4 Department of Geography and History, Prehistory Area, University of La Laguna, 38200 San Cristóbal de La Laguna, Spain

**Keywords:** Biochemistry, Zoology, Environmental social sciences

## Abstract

This paper seeks to reconstruct the management of food resources in the early Neolithic site of Cabecicos Negros in southeastern Spain. For this purpose, we have studied 29 potsherds from Cabecicos Negros (Andalusia, Spain). Applying the methods of gas chromatography and mass spectrometry we were able to recompose the daily use of the sherds related to the consumption and storage of food products. Among the results obtained in this work, we were able to show new evidence of the exploitation of dairy products in the south of the Iberian Peninsula, as well as provide information on the exploitation and management of the early domestic animals herds. To improve the archaeological results obtained, isotopic results were compared with a modern reference of 53 fat samples from the adipose tissue of domestic pigs and wild boars.

## Introduction

Organic residue analysis of lipids preserved in archaeological pottery vessel offers an alternative approach for determining the types of foods commonly consumed and processed in the past^1,2^, as adipose fats^3^, dairy products^4^, plants^5^, waxes^6^ or marine resources^7^. These studies have provided complementary evidence derived from zooarchaeological remains on animal management^8–12^.

The adoption of domestic animals at the beginning of the Neolithic was very variable in Europe, with different rhythms and patterns being documented^3,13–17^. In the Iberian Peninsula the practice of hunting activities was reduced with the introduction of the four domestic species (*Ovis aries*, *Capra hircus*, *Bos taurus* and *Sus domesticus*) between 5600 and 5400 cal BC, with a relative frequency averaging about 30% at peninsular level^15,16^. Wild boar and deer are the only species that maintain their overall importance in the diet during early Neolithic^16,18,19^. The high abundance of animal fats detected in ceramic vessels is consistent with the faunal assemblages and reflects the presence of animals in the diet. Investigations of early Neolithic pottery in Iberian Peninsula^20–28^ have revealed that ruminant (40%) and non-ruminant (20%) carcass fats were prevalent in pottery vessels with little evidence for the exploitation of dairy products (less than 10%).

Pig remains are usually scarce in the peninsular sites of the early Neolithic (less than 20% of sites exceed 100 remains)^16,29^, despite the fact that it was already widespread throughout the Iberian Peninsula by the middle of the sixth millennium BC. This specie present the problem of its differentiation from the wild boar^15^. Biometric studies on bone remains of wild and domestic pig show a great variability in size during early Neolithic, with a slight reduction in the average size of the pigs between early (5600–4500 cal BC) and middle Neolithic period (4500–3500 cal BC), supporting diverse pathways of domestication and adoption^29^. Archaeozoological methods have traditionally been used to detect pig exploitation in antiquity^12,30^. However, the ability to detect pork product processing and consumption directly from the vessels in which they were processed, offers new opportunities for linking the occurrence of residues to other cultural phenomena^31^. The way to observe wild and domestic animal species exploitation directly from organic residues preserved in pottery vessels provides a potentially valuable proxy at sites where animal bones are poorly preserved^12^. Because the isotopic compositions of food and fluids ingested by animals have a strong influence on the isotopic compositions of the tissues they synthesize^32^, the use of stable carbon isotope analyses in archaeological investigations allows research on animal metabolism and nutrition^33,34^. Moreover, variation may arise particularly in non-ruminant domesticates due to food supplements (e.g. from domestic waste such as whey left over from cheese production or meat scraps) which would contribute in a larger protein component perceived in the diet^32^. This process may be an indicator of certain livestock practices on pig that may indicate husbandry in the Neolithic period. However, very few studies have been carried out with a small number of reference samples to clearly differentiate between domestic and wild species^23,35,36^, although all of them show more impoverished isotopic values in those corresponding to wild taxa with respect to domestic species.

In the south of the Iberian Peninsula the available data on subsistence practices for the early Neolithic are relatively scarce but it is known that the percentage of domestic and wild fauna follows a similar trend to the one shown in the rest of the Iberian Peninsula^15^. The remains of domestic pigs are documented between 10 and 15% of the fauna represented, as in the Cueva de El Toro and Nerja^19,37,38^. However, lipids detected into the ceramic assemblage from Cueva de El Toro indicate that the presence of adipose pork fat in the vessels reaches 54%^28^. This overrepresentation of pork fat may respond to specific food processing or storage practices^27^. It is therefore necessary to expand the number of studies in this region in order to provide new data that provide a glimpse of the animal management and dietary practices carried out during the Neolithic.

In the present study, the analysis of 29 ceramic vessels from the Cabecicos Negros (Almería, Spain) site is proposed. Cabecicos Negros is an open-air Neolithic site located in the southeast of the Iberian Peninsula, facing the sea and near the mouth of the river Antas (see SupMat1). Due to the changes in temperature over the seasons and the taphonomic processes that open-air sites undergo, the preservation of organic remains is poor. Faunal and botanical remains are under-represented, making it difficult to know about animal and plant management strategies at this site. From the characterization of the products contained in pottery vessels by gas chromatography (GC), gas chromatography–mass spectrometry (GC–MS) and gas chromatography–combustion–isotope ratio mass spectrometry (GC–C–IRMS), we propose to highlight the isotopic breadth arising from different environmental/dietary/animal management regimes, taking into account the little information offered by the faunal remains. The application of organic residue analysis of pottery sherds in this paper provides new insights into the relationships between humans, animals and their environment.

Reliable classification of commodities processed in archaeological vessels can be made by comparing chemical structures of individual compounds with those obtained for modern reference materials^11^. However, it is assumed that at a particular location animal raised in antiquity would have consumed relatively restricted diets. Based on this assumption, the dietary contribution of δ^13^C values to tissues such as adipose fat would be relatively constant^32^. Therefore, in this work, 51 modern suids samples have also been analysed by GC–IRMS in order to provide for the first time a reference collection of domestic pig and wild boar. This methodological contribution allows evaluating to what extent it is possible to differentiate between hunting and livestock from the parameter of feeding and management of the animals.

## Materials

### Archaeological materials

For the chemical analysis of the residues in Cabecicos Negros (Vera, Almería), samples of 29 ceramic potsherds have been selected. Due to the high degree of fragmentation, it has not been possible to carry out a typological classification of the pottery assemblage, with a few exceptions. These cases are characterised by conical bottoms, convergent walls and a volumetry ranging between 10 and 15 L. However, a series of ceramic groups defined by the variety of printed, incised or plastic decorative motifs were documented, among which ceramics with cardial printed decoration stand out^39–42^.

The selection criteria for the 29 vases on a total corpus of 1902 vases (MNI) identified at the site of the Cabecicos Negros sought to ensure that the vases were representative of the different vessel morphologies. Given the high level of fragmentation of the ceramic assemblage, priority was given to those fragments in which it was possible to identify the provenance and intuit the shape of the vessel.

### Reference materials

In order to obtain reference isotopic values comparable with archaeological data from ceramic vessels, adipose tissue from modern animals have been selected. Samples of animal fat were obtained from a meat production slaughterhouse. The following aspects have been considered in the selection of the samples that make up this study.

**Figure 1:**
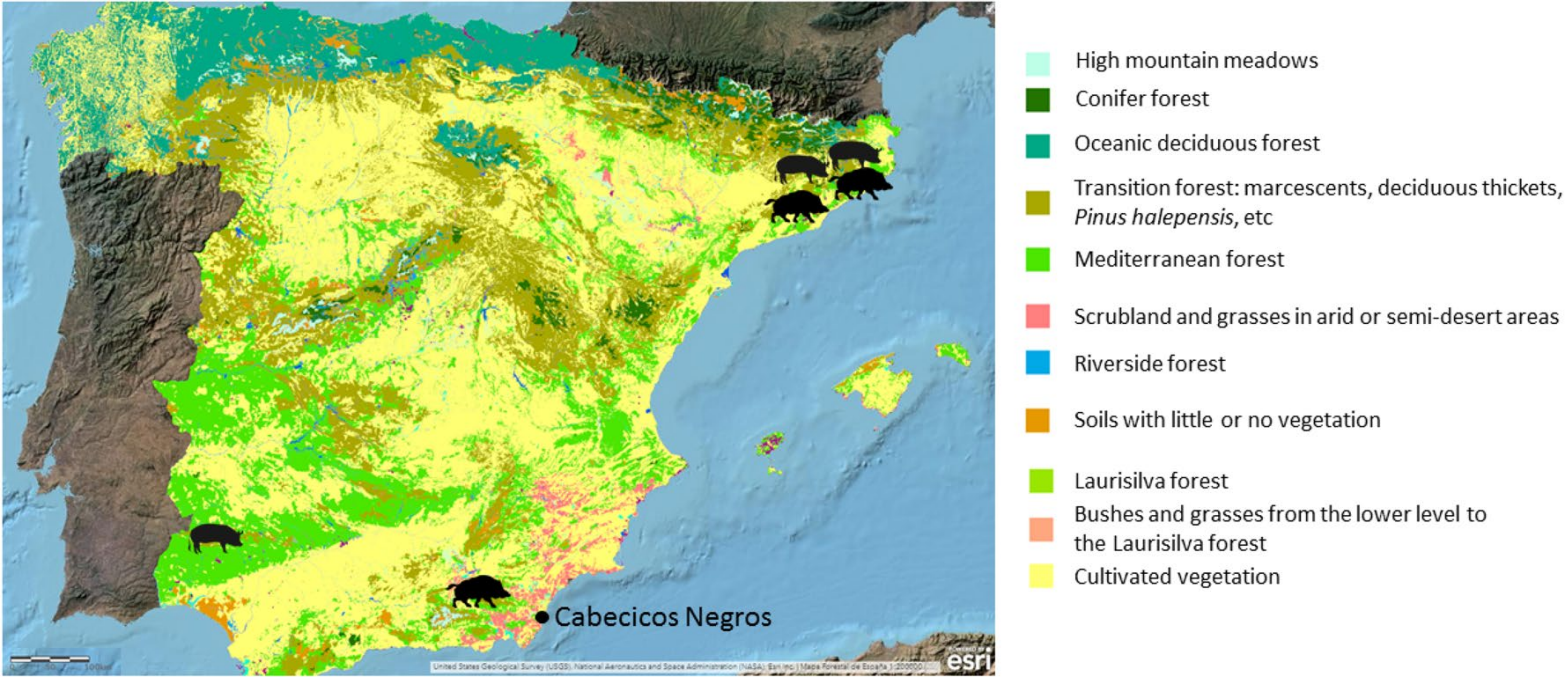
Vegetation map of the Iberian Peninsula with the location of the reference subspecies and the Cabecicos Negros site (https://www.arcgis.com/apps/Viewer/index.html?appid=c4c060eddfd447af833cdbf439015850#!).

Firstly, a large number of samples of each of the subspecies represented have been selected. A total of 53 reference samples were analysed in order to replicate the results, among them we find 26 domestic pigs and 27 wild boars from the Iberian Peninsula (Fig. 1).

The diet of the individuals was a condition for their selection. In monogastric animals, such as pigs and wild boars, digestion and absorption begin when fats from the diet reach the small intestine^43^. Dietary triglycerides enter the small intestine from the stomach and mix with secretions of bile and pancreatic juice in the duodenum. Bile salts allow emulsification of lipids, while pancreatic lipase hydrolyzes triglycerides to free fatty acids^44^. The mycelium that is formed allows hydrolyzed compounds to dissolve in intestinal contents, as well as other water-insoluble compounds such as fat-soluble vitamins and cholesterol. These can then be absorbed by the mucosal cells, where the fatty acids are resynthesized into triglycerides before passing to the lymph^44^.

One of the factors taken into account when selecting the animals was the absence of corn in their diet. Corn is a product originating from the American continent that appeared in Europe from the sixteenth century onwards, is often used to supply the herd during the winter or as a complement to the feeding on the pastures. In contrast, during the European Neolithic period, this type of plant was not part of the herd feed but was only part of the C3 type vegetation^45^. For this purpose, we contacted organically certified producers in various parts of the Iberian Peninsula who, in addition, did not include maize or other C4 plants in the animals' diet. The pigs have a lactation period of between 40 and 60 days and are fed a fodder composed of organic cereals: flour, barley, rye and wheat (GMO free). Domestic pigs can be separated into two groups according to their diet: a fodder-based diet rich in protein, and a semi-freedom diet based on acorns, grasses, legumes and wild fruits. Concerning wild boars, feeding is not controlled but comes from natural areas. The animals are from southeastern Iberia's dehesa and northeastern Iberia's Mediterranean forests, both consisting of *Quercus* and *Pinus*. In both cases, the most analogous situation to the prehistoric context that we could find in the peninsular region was sought (Supplementary Material, STable 1).

The age of animals affects the fat composition, e.g. C_18:0_ decreases over time^46^ or subcutaneous samples show an increase of C_18:1_ versus a decrease of C_18:0_ in older animals^47–49^. Although there is no direct affectation with the δ^13^C values. Animals have been selected according to the times of herd slaughter (when the animals achieve their optimum meat). The effects of breed and sex on fatty acid composition are relatively negligible^50,51^. Females had higher Δ^15^N values than males because males grew larger, whereas Δ^13^C values did not differ between sexes^51^.

**Figure 2:**
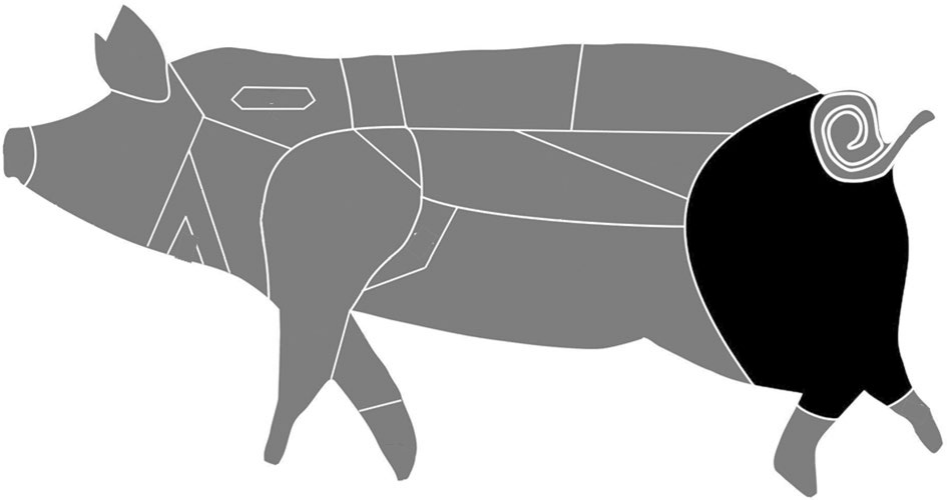
Pork cutting and processing areas and the area selected for the sampling of the reference collection (black).

The difference between the δ^13^C values of the fatty acids varies depending on the fat deposit from which it is extracted. The components are synthesised in different parts of the body, such as adipose fat, liver, and mammary gland, resulting in varying degrees of isotopic discrimination in fat synthesis^43,47,52^. The adipose fat of the back leg has been selected in all cases (Fig. 2), since the fat route is more direct and more representative of the animal diet^53^.

## Methods

### Archaeological sample extraction

All solvent extracted samples were analysed at the ICTA-Autonomous University of Barcelona. Lipids were extracted following published protocols^54^. The surface was cleaned with manual modelling drill to remove exogenous lipids, a 2 g fine powder was extracted with the cleaned drill. A volume of 50 µL of a hexane solution containing an internal standard (*n-*tetratriacontane*,* Sigma; 24.98 ng/µL) was added to the powder. Lipids were extracted by ultrasonication (2 × 15 min) using 4 mL of MeOH. Fatty acid methyl ester derivatives (FAMEs) have been prepared by adding 200 μL of sulphuric acid (H_2_SO_4_) maintained at 70 °C for 4 h. After cooling, water (3 mL) has been added and the lipids were extracted with cyclohexane (3 × 2 mL). The solvent was evaporated under nitrogen in readiness for GC analysis.

### Modern reference sample extraction

Ca. 5 g of modern animal fats, were taken with a scalpel from the ham fat of the selected animals using nitrile gloves. The sample was stored and frozen. A subsample of ca. 0.1 g is taken for extraction.

After adding 3 mL of sodium hydroxide (NaOH) (5%, 2 M in methanol), the mixture is heated in a heating plate to 70 °C and after 1 h it is left to cool. Next, a liquid–liquid extraction is made, shaking it with the help of a vortex and discarding the hexane phase with a Pasteur pipette to eliminate contaminating compounds and traces of H2O. To acidify the lipids, hydrochloric acid (HCl) (0,5 M, 32%) is added to obtain a pH3 and TLE is extracted (3 × 3 mL hexane).

A 2 mL of a mixture of 40 mL of MilliQ water and 500 μL of sodium chloride (NaCl) is added. Then, the hexane phase is extracted. The solvent is evaporated under a soft ray of nitrogen and, once dry, the acidified fraction is derivatised with 500 μL of a boron trifluoride complex with methanol (BF3-methanol) and heated in a heating plate to 75 °C for 1 h.

Once cooled, the reaction is stopped by adding 3 mL of MilliQ water and the lipids are extracted adding 3 mL of hexane. The hexane transferred to vial is evaporated under nitrogen and we add 50 μL of isooctane to inject the sample in the GC–IRMS to know the δ^13^C value of the released fatty acids.

### Instrument analysis

The gas chromatographic analyses were performed on an Agilent 7820A Gas Chromatograph fitted with a Flame Ionisation Detector (FID) using a DB-5 MS column (30 m length × 0.25 mm internal diameter × 0.25 μm stationary phase thickness). The splitless injector temperature was set at 300 °C and helium was used as the carrier gas. The temperature of the flame ionisation detector (FID) was 340 °C. The oven temperature was programmed to be held at 50 °C for 2 min, then the temperature increased at 15 °C/min to 170 °C, and finally to 320 °C at 6 °C/min, and held for a further 46 min. Data were acquired and processed using Agilent Chemstation software (v10, www.agilent.com).

The gas chromatography–mass spectrometry analyses were carried out using an Agilent 7890A Gas Chromatograph (GC) coupled to an Agilent 5975C Mass Spectrometer (MS). The GC was fitted with a DB-5 MS column (30 m length × 0.25 mm internal diameter × 0.25 μm stationary phase thickness). The GC injector was operated in splitless mode and helium was used as the carrier gas. The temperature of the flame ionisation detector (FID) was 320 °C. The oven temperature was initially held at 50 °C for 2 min, then the temperature increased at 15 °C/min to 170 °C, and finally to 320 °C at 6 °C/min, and held for a further 46 min. The Mass Spectrometer was run in electron impact mode and masses were acquired in full scan mode between *m/z* 50 to *m/z* 800. GC–MS instrument control, data acquisition was performed using the Masshunter Workstation software (v10, www.agilent.com). GC–MS data processing and graphic representation was performed using the Qualitative Analysis Module of the Masshunter Workstation (v10, www.agilent.com). Mass spectral interpretation was performed by comparison with the NIST/EPA/NIH Mass Spectral Library (version 2008, www.nist.gov/srd).

In order to determine the compound-specific stable isotopic determination (C18:0 and C16:0), a third analysis is performed using an isotope ratio mass spectrometer (IRMS) (Delta V, Thermo Fisher Scientific) hyphenated to a gas chromatograph (Trace GC, Thermo Fischer Scientific) via a combustion interface. The GC is fitted with a DB-5 MS-UI (60 m × 0.25 mm × 0.25 μm) column. The injector temperature is set at 310 °C. The oven is initially held at 80 °C for 1 min, then ramp at 30 °C/min to 120 °C, and finally increased to 320 °C at 6 °C/min and held for 21 min. Helium is used as the carrier gas. The combustion reactor is set at 940 °C. The samples were analysed in triplicate. GC-IRMS instrument control, data acquisition, data processing, quantitation, and graphic representation were performed with the Isodat v3.0 software (ThermoFisher Scientific, https://www.thermofisher.com). Analytical accuracy is confirmed by running fatty acid methyl ester (FAME) and alkane standards of known isotopic values prior to each batch of analysis. During each run, three pulses of carbon dioxide of known isotopic composition are fed into the ion source from the reference gas injector. These measures ensured that the instrument and combustion furnace are functioning properly. Instrument precision is ± 0.3‰.

The carbon isotope ratios are expressed relative to the standard reference material *v*PDB, δ^13^C ‰ = [R_sample_ − R_standard_]/R_standard_. The δ^13^C values were corrected for carbon atom(s) of the methyl group added during methylation of the fatty acids using the following equation: δ^13^C_FA_ = ((n + 1) × δ^13^C_FAME_) − δ^13^C_MeOH_)/n, where δ^13^C_FA_ is the corrected value for the fatty acid, n is the carbon chain length. Correction factor was obtained by derivatising a known δ^13^C_FA_ value with the derivatisating agent.

The δ^13^C measurements for C_16:0_ and C_18:0_ of the modern samples were also corrected for the post-Industrial Revolution effects of fossil fuel burning, which were found to have decreased the δ^13^C of atmospheric CO^2^ by 1.8‰^55^. To correct the change in the carbon isotopic signature (δ^13^C) of recent years, 1.14‰ was added to the δ^13^C values of the samples measured.

## Results

### Archaeological samples

Lipids from 29 potsherds were extracted by an acidified methanol extraction (Table 1). The preservation of lipids in association with pottery from Cabecicos Negros was less than other regions in Iberia^22^, with 65.5% of the samples yielding amounts of lipids above the accepted threshold of interpretation (i.e. > 5 μg/g)^56^. 19 of the 29 archaeological vessels examined from Cabecicos Negros contain biomarkers that identify the contents. The percentage of recovery appears to be affected by the open-air typology of the settlement as well as the length of time they were stored after exhumation.

**Table 1:** Organic residues analysis from Cabecicos Negros results.

Samples	TLE (µg g^−1^)	Lipid detected	δ^13^C		Predominant commodity type
			C16:0 ± 0.3 (‰)	C18:0 ± 0.3 (‰)	
CNP01	86.9	FA (14, 16 < 18)	− 28.5	− 27.5	Non-ruminant adipose fat
CNP02	51.3	FA (16 > 18, 18:1)	− 27.9	− 27.2	Non-ruminant adipose fat
CNP06	91.4	FA (16 > 18)	− 27.5	− 25.7	Non-ruminant adipose fat
CNP12	896.65	FA (16 < 18)	− 26.8	− 31.9	Dairy fat
CNP13	65.2	FA (14, 16 > 18)	− 26.1	− 25.1	Non-ruminant adipose fat
CNP14	66.8	FA (16 > 18, 18:1)	− 24.3	− 24.6	Non-ruminant adipose fat
CNP15	87.1	FA (16 > 18, 18:1); Alkanes: C27–C37	− 27.6	− 25.4	Non-ruminant adipose fat
CNP16	95.4	FA (16 > 18)	− 26.2	− 24.6	Non-ruminant adipose fat
CNP17	69.3	FA (16 > 18)	− 23.9	− 22.8	Non-ruminant adipose fat
CNP18	88.9	FA (14, 16 > 18)	− 26.3	− 27.8	Ruminant adipose fat
CNP19	43.5	FA (14, 16 > 18)	− 26.7	− 25.5	Non-ruminant adipose fat
CNP21	132.45	FA (14, 16 > 18, 20)	− 28.4	− 25.3	Non-ruminant adipose fat
CNP22	21.8	FA (16 > 18)	− 27.9	− 26.0	Non-ruminant adipose fat
CNP23	84.3	FA (16 > 18, 18:1)	− 25.4	− 25.7	Non-ruminant adipose fat
CNP24	74.2	FA (16 > 18)	− 27.9	− 26.7	Non-ruminant adipose fat
CNP26	39.5	FA (16 > 18)	− 25.5	− 24.1	Non-ruminant adipose fat
CNP27	18.1	FA (14, 16 > 18)	− 26.2	− 25.8	Non-ruminant adipose fat
CNP28	23.6	FA (16 > 18)	− 26.8	− 26.3	Non-ruminant adipose fat
CNP29	49.5	FA (16 > 18, 18:1); Alkanes: C27–C37	− 27.5	− 25.9	Non-ruminant adipose fat

*FA* fatty acids, *TLE* total lipid extract.

**Figure 3:**
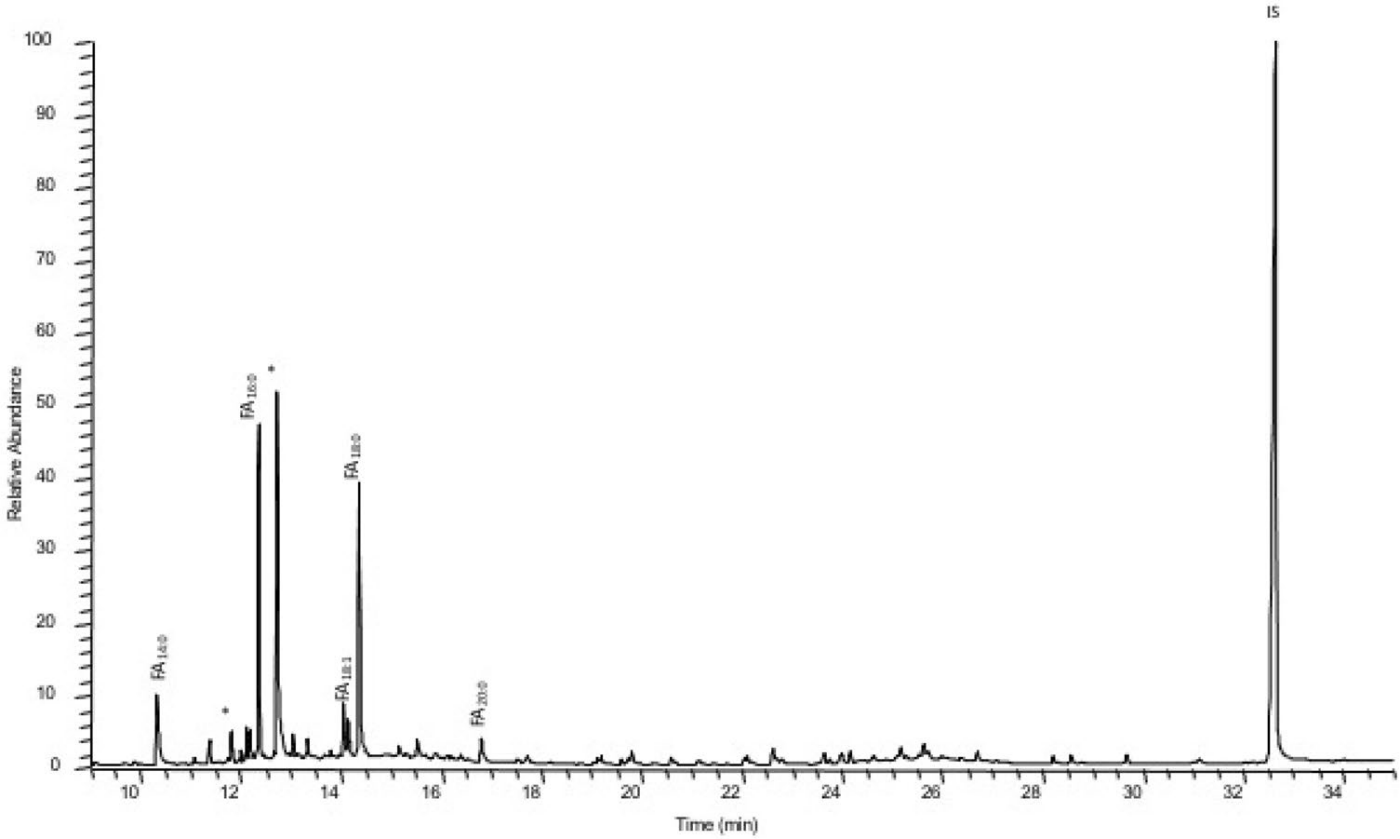
Partial gas chromatogram of total lipid extract of sample CNP21; FAn:0: saturated fatty acids with n carbon atoms; IS: internal standard, C34 *n*-tetratricontane; *contaminants (phthalates).

Cabecicos Negros potsherds revealed a range of saturated and unsaturated mid-chain length *n-*alkanoic acids (fatty acids) with even numbers of carbon atoms from C_14:0_ to C_20:0_, particularly dominated by C_16:0_ and C_18:0_ (Fig. 3). Odd-numbered fatty acids (C_17:0_), biomarkers of bacterial population from the rumen and characteristic of ruminant fats^57,58^, was detected in CNP18 sample. The relatively high abundance of the palmitic acid (C_16:0_) compared to the stearic acid (C_18:0_) in 84% of the extracts suggests that these lipids could derive from non-ruminants adipose fats^59^. Low concentrations of oleic acid (C_18:1_) were found in 4 samples (21%). There are different sources of origin of C_18:1_: plant lipids^58^, modern contamination^9^ and it can be found in animal fat triacylglycerols^60,61^. In 2 extracts (10,5%) n-alkanes (C_27_–C_37_) were also detected and could originate from higher plants^60^. However, the absence of plant sterols does not allow us to interpret a plant input. No marine resource biomarkers, such as isoprenoid acids (4,8,12-trimethyltridecanoic acid, phytanic or pristanic acid), ω-(*o*-alkylphenyl)alkanoic acids (APAAs) or long fatty acid chains with more than 18 carbon atoms, were detected in any of the samples analysed^56^. The presence of phthalates was found in all of the analysed samples. This contamination might come from the plastic bags in which the vessels were stored.

**Figure 4:**
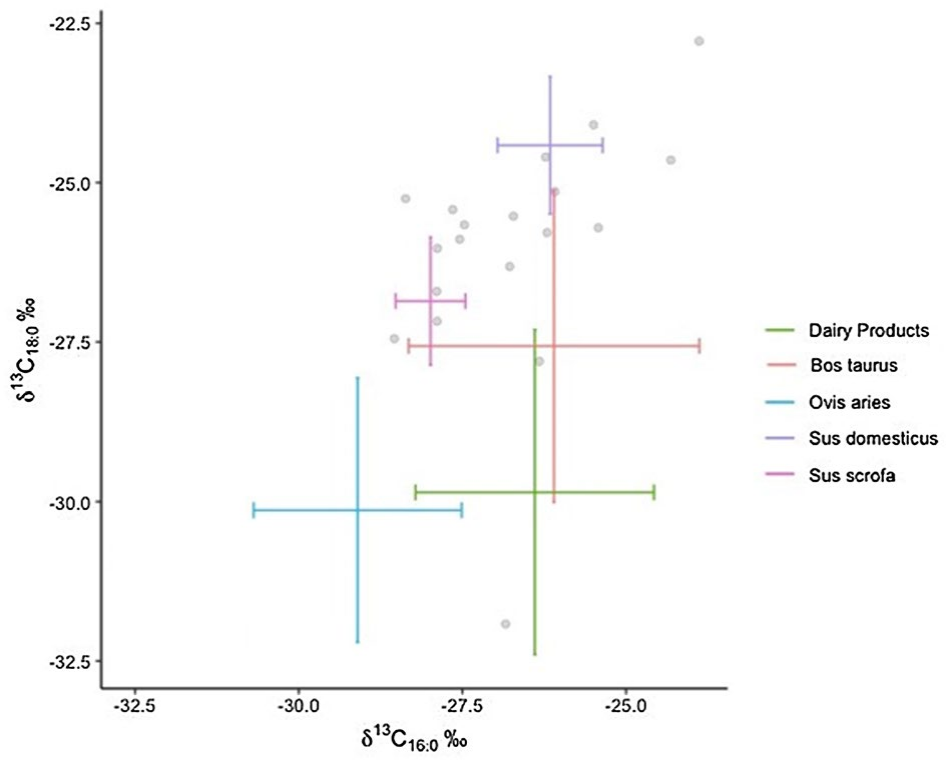
Scatter plot showing δ^13^C_16:0_ (*X*) and Δ^13^C values (*Y*) of fatty acids extracted from Cabecicos Negros site compared with δ^13^C values from modern reference animal fat (Supplementary Material, STable 2).

Identification of the source of the degraded animal fats recovered from the pottery (n = 19) took place through the determination of the carbon isotopic composition (δ^13^C) of the C_16:0_ and C_18:0_ fatty acids (Fig. 4). The δ^13^C16:0 values of archaeological animal fats ranged between − 28.5 and − 23.88‰, while δ^13^C_18:0_ values range between − 31.9 and − 22.8‰. Results were compared with fatty acid δ^13^C values from modern reference adipose tissue^23,57,62–64^ (Supplementary Material, STable 2). These δ^13^C values are in agreement with pure fats and mixtures of carcass fats from non-ruminant animals, ruminant animals and dairy products.

Over 90% of the archaeological animal fats extracted from the pots from Cabecicos Negros exhibit Δ^13^C values consistent with non-ruminant adipose fat. Although values corresponding to ruminant adipose fat (CNP18) and dairy product fat (CNP12) have also been identified. Considering the estuarine location of the site, we cannot rule out the possibility that marine resources were processed in the vessels. In fact, some CNP14 and CNP17 δ^13^C values appear to be more enriched than the rest of the non-ruminants. These values could also be the result of product mixing or non-ruminants being fed marine products.

### Reference samples

From the 53 samples analysed from three types of suid populations (corrected values), an average of − 22.5‰ isotopic values of palmitic acid (δ^13^C_16:0_) with a standard deviation (SD) of 1.6‰ for domestic pigs fed with fodder, − 24.5‰ with a SD of 1.7‰ for pigs fed with acorns, grasses, legumes and wild fruits, and − 26.6‰ with a SD of 2.6‰ for wild boars. Stearic acid isotopic values (δ^13^C_18:0_) in domestic pigs fed with fodder are − 20.6‰ with a SD of 0.8‰, − 23‰ with a SD of 1.6‰ in pigs fed with acorns, grasses, legumes and wild fruits, and − 26.3‰ with a SD of 3.01‰ for wild boars (Supplementary Material, STable 3).

**Figure 5:**
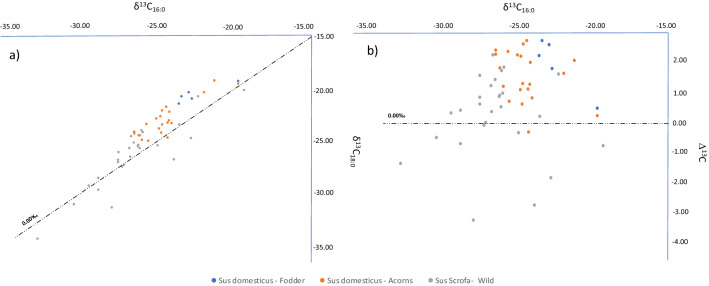
(**a**) Scatter plot showing δ^13^C_16:0_ values (X axis) and δ^13^C_18:0_ values (Y axis), and (**b**) δ^13^C_16:0_ values (X axis) and Δ^13^C (= δ^13^C _18:0_ − δ^13^C_16:0_) values (Y axis) of fatty acids extracted from Pigs (*Sus domesticus*) with a fodder-based feed (blue); fed by acorns, grasses, legumes and wild fruits (Orange); and Wild boars (*Sus scrofa*) (grey).

These data allow us to identify three distinct *S.* sp. populations based on the type of feed received. As shown in Fig. 5a and b, the three populations can be distinguished by the δ^13^C values of palmitic acid (C_16:0_), with *S. domesticus* fed with fooder having higher values than *S. scrofa* fed wild.

## Discussion

The few faunal samples corresponding to early Neolithic sites studied in the southern peninsular sites highlight the importance of ovicaprines^15,37^. The present study has made possible to document the processing in sherds of ruminant and non-ruminant fats and the dairy exploitation. These results allow us to demonstrate the exploitation of ruminant and non-ruminant species. Furthermore, milk identification indicates that dairy production has occurred and that consequently, the presence of goats, sheep or cows among the ruminant species has been confirmed.

It is also worth discussing the identification of non-ruminant fats in sherds from Cabecicos Negros. The presence of suids in the faunal record is estimated to be around 23.9% and varies considerably between sites and throughout the Neolithic periods, suggesting different scales of livestock regimes^15,16,65^. The suids would have been a valuable product for prehistoric people; they have a short reproductive cycle of 21 days and gestation of 112–126 days^66^. The litter size ranges from 3 to 5 offspring, so under favourable conditions, they have an extraordinary reproductive capacity^15,67^. Swine feeding habits are generally opportunistic, which has caused pigs to eat virtually anything. They can be fed through human waste, providing means of controlling settlement garbage^68^.

Previous studies on lipid residues extracted from prehistoric ceramics in the Iberian Peninsula have shown that only a few vessels contain porcine lipids. The presence of non-ruminant fats in the Early Neolithic vessels of the Iberian Peninsula does not exceed 20%, being lower than 16% in the west^22^, around 19% in the north^22,25^ and around 18% in the northeast of the Iberian Peninsula^20,21,24,27,28^. At the moment, only one case study has showed the evidence of non-ruminant fat in vessels from the early Neolithic period in the south of the Iberian Peninsula. In Cueva de El Toro 54% of these fats were recorded in the total number of vessels analysed with a lipid presence^28^. This low record of pork fat in vessels from other peninsular regions may be due to alternative methods of cooking pork which exclude the use of pottery, such as roasting^69^. There might also be a dietary bias, whereby beef, lamb and dairy products were cooked and consumed in preference to pork^12^. The overrepresentation of pork fats in Cueva de El Toro pottery could respond to non-food purposes, such as perishable products preservation^28^, or to a livestock practice focused on pig farming in this region of Iberia.

**Figure 6:**
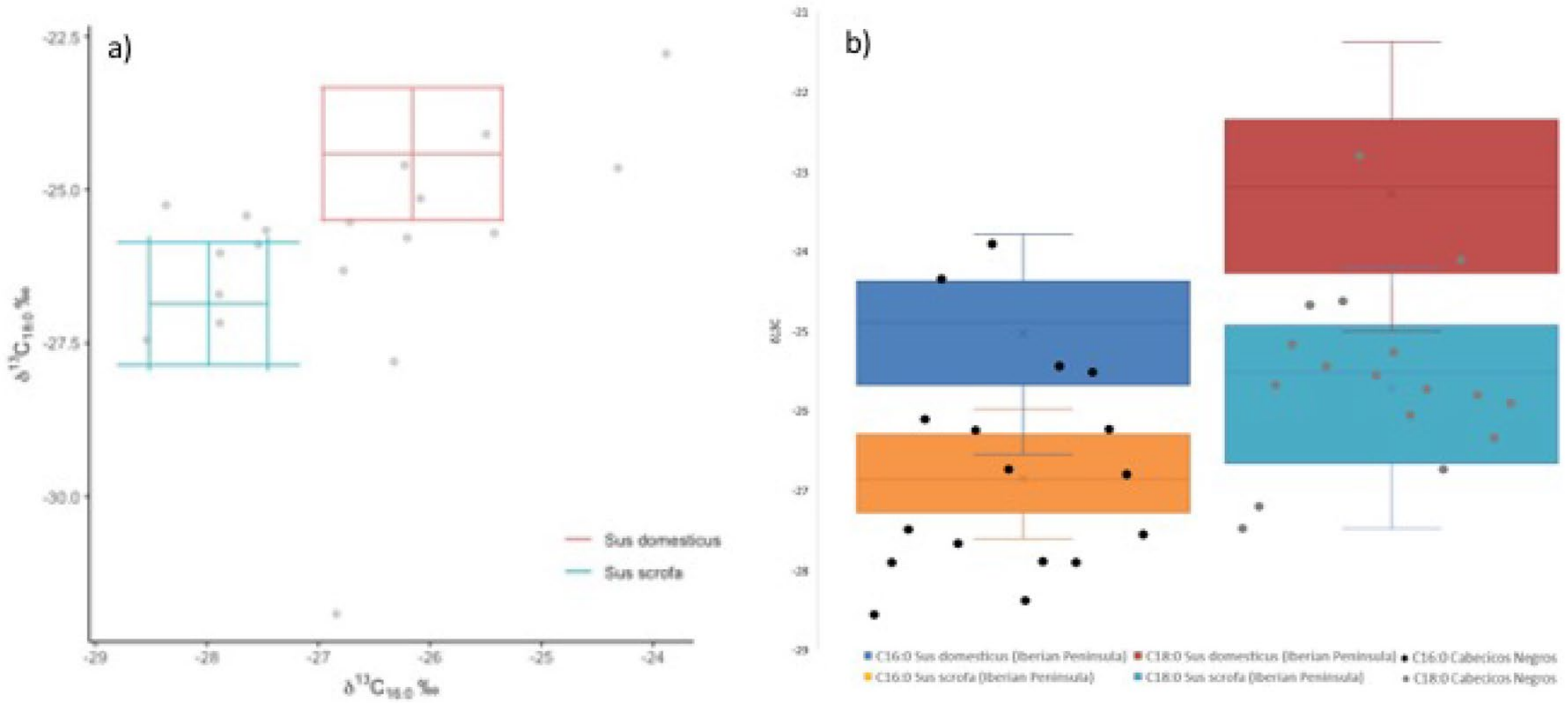
(**a**) Scatter plot showing δ^13^C_16:0_ (X) and δ^13^C_18:0_ (Y) values of fatty acids extracted from Cabecicos Negros site pottery compared with the modern reference fats from pigs and wild boars. (**b**) Boxplot with the mean values of domestic pig and wild boar fats from the Iberian Peninsula and the isotopic values from Cabecicos Negros fats.

The comparison of compound isotopic (δ^13^C) results of fatty acids extracted from Cabecicos Negros pottery, between modern wild boar and modern domestic pig fats has been able to distinguish the origin of the identified fats (Fig. 6a,b). Modern fat reference collections currently suggest a distinction between *Sus domesticus* and *Sus scrofa*, though literature on wild animal fats remains scarce^23,35,63^. From modern values obtained with the 27 samples of wild boar adipose fats and the 26 samples of pig adipose fat, the archaeological results from Cabecicos Negros are consistent with the ellipse of modern pig adipose fats, as well as with the values for wild boar adipose fats (Fig. 5).

The wide range of C_16:0_ and C_18:0_ values between non-ruminant samples in Cabecicos Negros, which differ between domestic pigs (*Sus domesticus*) and wild boar (*Sus scrofa*), leads us to think that the diet could reflect this isotopic differentiation in the fats detected in Cabecicos Negros. Even though the distribution of non-ruminants detected in archaeological vessels from the Neolithic site of Cabecicos Negros follows a normal distribution (Shapiro–Wilk, *p* = 0.71), the extremes within the population may respond to a differentiated diet.

These variations in δ^13^C values between domestic and feral pigs are also noticeable in enamel sequences^70^. The degree of animal protein contribution in pigs may reflect the degree of control and type of livestock management carried out^29,71–76^. We must bear in mind that isotopic variation between the reference values of domestic pig and wild boar fats does not respond to a differentiation between species but rather a variation in diet. The existence of complex scenarios for the initial domestication and rearing of pigs leads to adopting approaches that go beyond the wild-domestic dichotomy. The adoption and rearing of domestic pigs varied, resulting in a continuous crossover between domestic and feral taxa^29^ during the early Neolithic. Different degrees of intensity of the relationship between pig and human societies are also considered^71,77^.

As a result, a distinction in *Sus* sp. management in Cabecicos Negros should be considered. In contrast to the enrichment of the value of carbon in Neolithic pigs, other works propose that this change may be due to different factors: feeding in open environments^78–80^, feeding linked to human waste, a marine contribution in the diet, or the consumption of mushrooms^78^.

Traditional breeding practises may have involved home-based systems with full or partial housing of herds near settlements or extensive management of herds in semi-freedom or open-air regimes. Although these management practises are known in modern traditional communities in northern Mediterranean^81^, they have also been proposed for prehistoric European groups^82^. Under a extensive management regime, oak and riverbank forests like those found around Cabecicos Negros are ideal for breeding wild boar or domestic pigs^83^. A recent study on feeding management strategies for domestic pigs at Neolithic sites in the northeast of the peninsula showed that there are differences in pig management between different sites based on the isotopic ratio of carbon (^13^C/^12^C) and nitrogen (^15^N/^14^N)^29^. On one hand, La Draga, Cova del Frare and Serra del Mas Bonet showed consistent values with their respective local herbivores and could be associated with an open-air husbandry system in forest environments^29^. On the other hand, Can Sadurní and Reina Amàlia-Caserna de Sant Pau presented values δ^15^N higher than the local herbivores by 2.5 and 1.4‰ respectively, and in the case of Can Sadurní similar to carnivore δ^15^N values. The authors interpret that these data could be due to selective feeding practices or the variability of protein intake^29^.

These results lead us to question the causes of the differentiation in diets within the same site, as seen in Cabecicos Negros:

- Complementarity of livestock and hunting. These study results could indicate the existence of pig livestock practices together with the hunting practices of wild boars in Cabecicos Negros site.
- Differentiation by sex or age. The changes in pig diet in Cabecicos Negros may respond to an intentional practice of greater control over these animals. The ethnographic data collected shows that the enclosure criteria may respond to reproductive reasons^84^. Under optimal feeding conditions, sows bred could give birth from the first year of life. In this way, some herders retain sows to continue lactating and encourage reproduction until 7–8 years of age, while others slaughter sows around 4–5 years before the meat becomes too hard^84^.
- Changes in livestock management. The widely disparate δ^13^C values in the pig population can respond to different livestock management practices: the occurrence and seasonality^64^ or the mobility of livestock in food search^85^. Animals would have modified their diet in response to seasonal periods or times of famine with available diferent resources.

In summary, we see the variability of the values δ^13^C corresponding to a direct contribution of the differentiated diet. The low δ^13^C in most non-ruminants of Cabecicos Negros may correspond to a diet in forested or riparian environments^86^. Plants and trees in dense forests are more depleted in δ^13^C value than in open grasslands, especially plants closer to the ground^82^. Thus, the observed low levels of δ^13^C in suids are consistent with a differentiation between extensive diets versus a more omnivorous dietary intake. In this sense, we have discussed the possible causes of pig dietary differentiation have been discussed, including mixed subsistence strategies that combine livestock and hunting activities, a more omnivorous diet in females reflecting an interest in reproductive control, and changes in herd diet due to occurrence, seasonality, or resource availability in Cabecicos Negros during the early Neolithic.

## Supplementary Information

Supplementary Information 1.

Supplementary Table 1.

Supplementary Table 2.

Supplementary Table 3.

## Data Availability

All data generated or analysed during this study are included in this published article and its supplementary information files.
