# Supplementary material for: Investigating livestock management in the early Neolithic archaeological site of Cabecicos Negros (Almería, Spain) from the organic residue analysis in pottery
Source: Sci Rep. 2023 Mar 23;13:4797. doi: 10.1038/s41598-023-31036-6 (PMC10036527; doi:10.1038/s41598-023-31036-6)
Supplement: Supplementary file 1 — Supplementary Information 1. [file 41598_2023_31036_MOESM1_ESM.docx]

**Supplementary Material. Cabecicos Negros site**

Cabecicos Negros (Vera, Almería) is an open-air Neolithic settlement located southeast of the Iberian Peninsula. It is currently located about two kilometres from the mouth of the river Antas and at an altitude of 20 metres above sea level (Fig. 1). However, during the Early Neolithic, its location would have been close to the coast, and its immediate surroundings would have consisted of a vast bay^1^. The available documentation derives from two archaeological interventions carried out in 1991 and 2000^2,3,4,5,6^. Together with the site known as El Pajarraco, this site forms a sizeable archaeological complex with an uneven occupation along the plain througout the Early Neolithic and the Roman periods^3^.


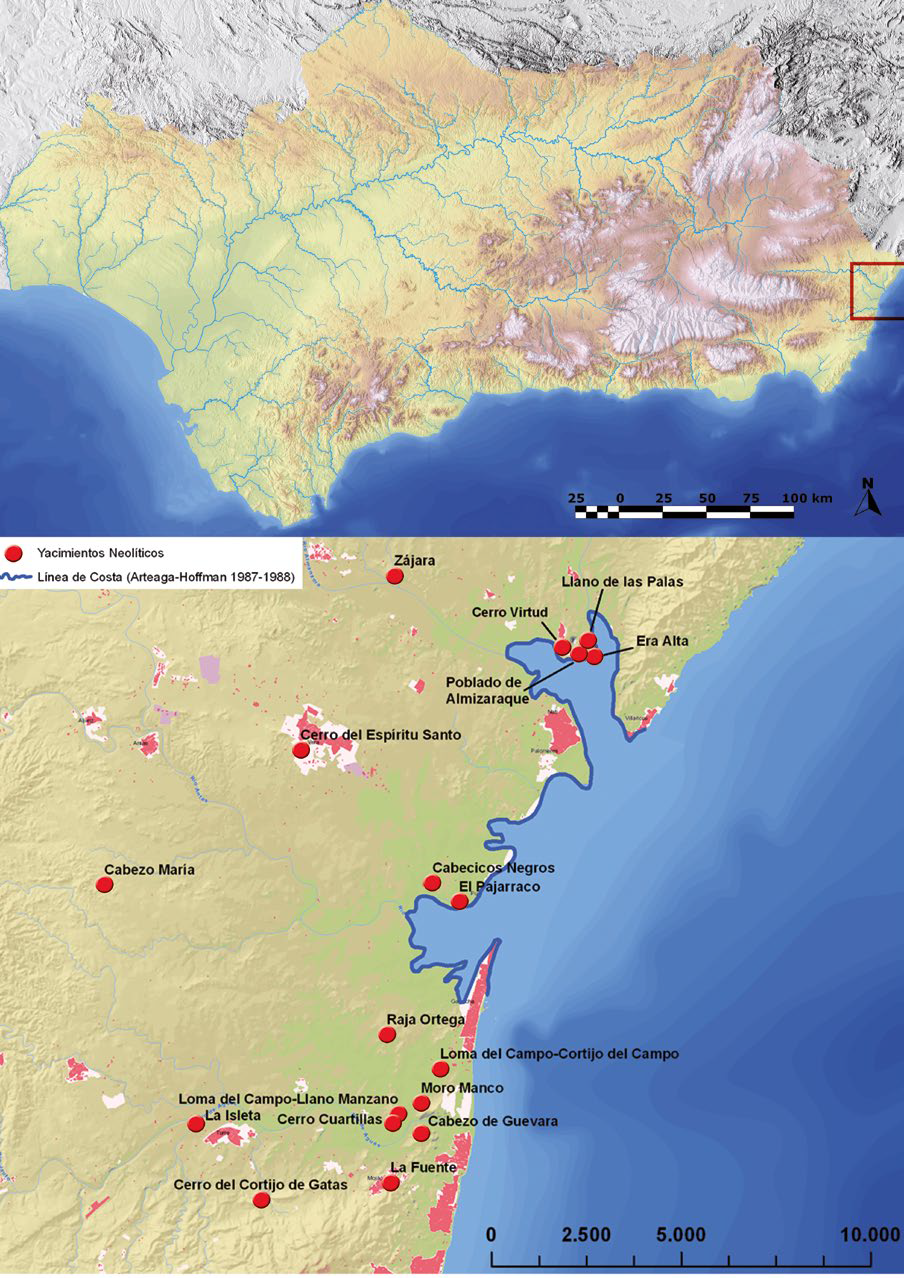


**Figure 1.** Geographical location of the Early Neolithic settlement of Cabecicos Negros and the Neolithic sites of the Vera depression and the Almanzora river basin. (Design: Juan Carlos Mejías García).

The evidence of occupation during the Early Neolithic is concentrated in the northwestern sector, configured by six small elevations arranged on the left bank of the river Antas, which constitute the preferred geomorphological units of habitation during this period^2,3,4^. The excavated area, located on the northernmost hill with an excavated surface of 100 m^2^, has provided four dates on Cerastoderma edule, which, together with the techno-morphological characteristics of the identified material assemblages, have allowed us to pinpoint a single occupation during the Early Neolithic^2,5,6^. Specifically, at an incipient moment of the emergence of the production economy in the southeast of the peninsula, marked by agricultural and livestock activities and craft diversification^2,6^.

Thus, it has been possible to recognise an occupation mainly related to exploiting of biotic and abiotic resources to produce personal ornaments made of stone and shell^5,7,8,9^. Among them, the production of lithic bracelets made of limestone and, above all, slate and necklace beads made of shell, stand out. The taphonomic characteristics of the malacofauna, preferably Cerastoderma and Glycimeris, suggest that they were collected alive in the site’s immediate surroundings. On the one hand, the small number of individualised marine shells and their meagre nutritional contribution suggest that this shellfish activity must have had little importance in the diet. On the other hand, the large number of ornamental elements made from mollusc shells link the primary function of this site to bromatological activities, i.e. a workshop for the manufacture of personal adornment^10^.

The technical procedures and the typology of the bracelets and necklace beads are highly standardised. Furthermore, a comprehensive representation of pieces in the transformation process has been documented, which has allowed an approximation to be made to the operational chain of this production^7^. In line with this, the functional analysis of the carved lithic production, with many drills on blades, has shown that a significant proportion of the sample was used on mineral materials (36%)^11^. Therefore, this is a settlement in which a specialised craft activity was carried out, with a production volume that must have exceeded the internal demand of the community. Thus, the presence of slate bracelets in areas of the Levante peninsular during the Early Neolithic has been related to an interregional circulation of this type of product from specialised settlements such as Cabecicos Negros^9^.

This economic orientation cannot be understood without considering that these communities would be characterised by an itinerant occupation in a broad territorial framework, where the seasonal/periodic habitation of this type of settlement would be essential for the capture and exploitation of the abiotic and biotic resources necessary for this specialised craft production^2,5,6^. This hypothesis is ratified by the small size of the habitational structures, the absence of those related to storage or the significant fragmentation and/or exhaustion of the material record. On the other hand, the evidence of the recurrent occupation of the different hills during the Early Neolithic could be explained by the need for this type of raw materials, which were abundant in the area, as they constituted the primary economy of these communities. Botanical and faunal remains are under-represented, making it difficult to understand site’s animal and plant management strategies at this site.

The pottery appears with a high rate of fragmentation that has not allowed for a typological classification of the assemblage, with a few exceptions. These cases are characterised by conical bottoms, convergent walls and a volumetry ranging between 10 and 15 litres. However, a series of ceramic groups defined by the variety of printed, incised or plastic decorative motifs were documented, among which ceramics with cardial printed decoration stand out^3,4,5,6^.

^1^ Arteaga Maute, O. & Hoffmann, G. Dialéctica del Proceso Natural y Sociohistórico en las Costas Mediterráneas de Andalucía. *Revista Atlántica-Mediterránea de Prehistoria y Arqueología Social*, 2, 13-121 (1999)

^2^ Camalich Massieu, M.D., Martín-Socas, D. & González Quintero, P. (eds.) *El territorio almeriense desde los inicios de la producción hasta fines de la Antigüedad* (Arqueología Monografías, 1999).

^3^ Goñi Quinteiro, A., Chávez Álvarez, E., Camalich Massieu, M.D., Martín Socas, D. & González Quintero, P. Intervención Arqueológica de Urgencia en el Poblado de Cabecicos Negros (Vera, Almería). Informe Preliminar. *Anuario Arqueológico de Andalucía 2000*, III, 73-87 (2002)

^4^ Camalich Massieu, M. D. *et al*. The Neolithic in Almería: The valley of the Almanzora river and Vera basin. *Documenta Praehistorica* 31, 183-197 (2004)

^5^ Camalich Massieu, M.D. & Martín-Socas, D. Los inicios de Neolítico en Andalucía. Entre la tradición y la innovación. *Menga Revista de Prehistoria de Andalucía*, 4, 103-129 (2013)

^6^ Martín-Socas, D., Camalich, M.D., Caro, J.L. & Rodríguez-Santos, F.J. The beginning of the Neolithic in Andalusia. *Quaternary International*, 470, 451-471 (2018) <https://dx.doi.org/10.1016/j.quaint.2017.06.057>

^7^ Goñi Quinteiro, A., Rodríguez Rodríguez, A.C., Cámalich Massieu, M.D., Martín Socas, D. & Francisco Ortega, M.I. La Tecnología de los elementos de adorno personal en materias minerales durante el Neolítico Medio. El ejemplo del poblado de Cabecicos Negros (Almería). *Actes del II Congrès del Neolític a la Península Ibérica. Sagvnvm-Plav*, Extra-2, 163-170 (1999)

^8^ Martínez-Sevilla, F. Los contextos de producción de brazaletes de piedra neolíticos en el sur de la Península Ibérica y sus implicacions socioeconómicas. *Actas del II Congreso de Prehistoria de Andalucía*, Sevilla, 317-328 (2014)

^9^ Orozco Köhler, T. Los brazaletes de esquisto: un elemento de la identidad cardial. In *Del neolític a l’edat del bronze en el Mediterrani occidental. Estudis en homenantge a Bernat Martí Oliver Bonet* (ed. Rosado, H.) 141-146 (TV SIP 119, 2004)

^10^ García-Escárzaga, A. & Rodríguez-Santo, F.J. Análisis arqueomalacológico y relaciones elementales Sr/Li en Cerastoderma edule (Linnaeus, 1758) del yacimiento neolítico de Cabecicos Negros (Vera, Almería), *Menga. Revista de Prehistoria de Andalucía* (*in press*)

^11^ Rodríguez-Rodríguez, A. Análisis funcional del instrumental lítico tallado del poblado de Cabecicos Negros. In *El territorio almeriense desde los inicios de la producción hasta fines de la Antigüedad. Un modelo: la depresión de Vera y cuenca del Almanzora,* 225-235 (Arqueología Monografias Junta de Andalucia, 1999)
